# Sexual antagonism in sequential hermaphrodites

**DOI:** 10.1098/rspb.2023.2222

**Published:** 2023-11-22

**Authors:** Thomas J. Hitchcock, Andy Gardner

**Affiliations:** ^1^ RIKEN Interdisciplinary Theoretical and Mathematical Sciences (iTHEMS), RIKEN, Wako, Saitama 351-0198, Japan; ^2^ School of Biology, University of St Andrews, St Andrews, Fife KY16 9TH, UK

**Keywords:** sequential hermaphroditism, protogyny, protandry, intragenomic conflict, intralocus sexual conflict

## Abstract

Females and males may have distinct phenotypic optima, but share essentially the same complement of genes, potentially leading to trade-offs between attaining high fitness through female versus male reproductive success. Such sexual antagonism may be particularly acute in hermaphrodites, where both reproductive strategies are housed within a single individual. While previous models have focused on simultaneous hermaphroditism, we lack theory for how sexual antagonism may play out under sequential hermaphroditism, which has the additional complexities of age-structure. Here, we develop a formal theory of sexual antagonism in sequential hermaphrodites. First, we construct a general theoretical overview of the problem, then consider different types of sexually antagonistic and life-history trade-offs, under different modes of genetic inheritance (autosomal or cytoplasmic), and different forms of sequential hermaphroditism (protogynous, protoandrous or bidirectional). Finally, we provide a concrete illustration of these general patterns by developing a two-stage two-sex model, which yields conditions for both invasion of sexually antagonistic alleles and maintenance of sexually antagonistic polymorphisms.

## 1. Introduction

Females and males may have distinct phenotypic optima, encompassing different behaviours, morphologies and physiologies [1,2]. However, the sexes, for the most part, share an identical complement of genes, and this can lead to trade-offs between attaining high fitness through each of these two routes, with alleles that are beneficial in relation to female reproductive success being costly in relation to male reproductive success, and *vice versa*. While the dynamics of sexual antagonism have long been studied [3–6], this was typically in a restricted set of conditions (e.g. panmixia, non-overlapping generations), and there has been an increased recent interest in how sexual antagonism may be expected to play out in a more diverse range of ecological and genetic scenarios. This has included: population structure and inbreeding [7–11], unusual modes of inheritance [11–13], age structure [9,14] and mechanisms of population regulation [15–17].

One group of organisms within which sexual antagonism may be particularly acute are hermaphrodites. In these organisms, female and male reproductive strategies are housed within a single individual, and thus they may experience more intense sexual antagonism than do dioecious species [18]. Previous theoretical work considering sexual antagonism in hermaphroditic organisms has primarily focused on simultaneous hermaphrodites—i.e. when individuals produce sperm and eggs concurrently throughout their life [8,17,19,20]. By contrast, sequential hermaphroditism—i.e. when individuals temporally separate the production of male and female gametes—has been relatively overlooked. Although there have been some verbal treatments [18,21,22], no formal theory has yet been developed on sexual antagonism under sequential hermaphroditism.

Yet sequential hermaphrodites exhibit many interesting features that may make them exceptional study systems for improving our understanding of sexual antagonism. Firstly, owing to the way in which female and male strategies are temporally stratified, age structure is likely to be an important modulator of evolutionary outcomes. Recent work has shown how sex differences in fecundity and mortality schedules may shape sexual antagonism in dioecious (gonochoristic) taxa [9,14]; thus sequential hermaphrodites, with their diversity of combinations of age and sex structure—from protandry (male first), to protogyny (female first) and bidirectional sex change [23]—may prove superb systems with which to test these principles, as not only may asymmetries in parental age matter, but also asymmetries in patterns of sex change. Yet we currently lack theory that allows for such life cycles, and thus with which to firmly base these suggestions.

Moreover, while in dioecious systems the flow of genes between sexes occurs solely through reproduction, in sequential hermaphrodites this also occurs through sex change. This may have profound consequences. For instance, males are typically an evolutionary dead end for mitochondrial genes in dioecious species, as they usually do not pass these genes on to their offspring, and mitochondria are therefore liable to accumulate male-deleterious mutations—the ‘mother's curse' [24,25]. By contrast, a male's mitochondria will have reproductive value insofar as he is able to change sex and produce offspring as a female later in life, such that male-deleterious mitochondrial alleles need not be invisible to selection in sequential hermaphrodites.

Here, we develop a formal theory of sexual antagonism in sequential hermaphrodites. We construct a general theoretical framework, which reveals how the reproductive values of males and females are shaped by transmission genetics, age structure, and patterns of sex change, and we use this to investigate how different sexually antagonistic and life-history trade-offs are modulated by differences in the mode of genetic inheritance (autosomal or cytoplasmic) and the form of sequential hermaphroditism (protogynous, protoandrous or bidirectional). We provide a concrete illustration of these general patterns by developing a two-stage two-sex model, yielding conditions for both the invasion of sexually antagonistic alleles and the maintenance of sexually antagonistic polymorphisms.

## 2. Reproductive value in age- and sex-structured species

When populations are divided into different classes, such as age and/or sex, then the weights upon selective effects in these different classes [26,27]—are given by their reproductive values, i.e. the expected asymptotic fraction of genes in future generations that will descend from that particular class of individuals in the current generation [28–32]. If transitions between classes are governed by an aperiodic Markov process that has attained its steady state behaviour (i.e. stable class distribution), then these contributions to the future are equivalent to the relative amounts of time that a gene's lineage will have spent in a particular state. If we assume that all newborns have the same independent probability of being male or female, then we may calculate a class's reproductive value simply by sampling a gene from a newborn and tracing its origin back to one of the newborn's parents, determine the total extent of the parent's life up to this moment that was spent as a member of this class, and then take the expectation of this quantity across all newborns (further derivation is given in the electronic supplementary material, S1 and S2).

Sampling a gene from a newborn individual, we notate the probability that it derived from the mother by *ζ*, and the probability it derived from the father by 1 − *ζ*. If it derived from the mother, then with probability *μ_a_* she is of age *a*, and if derived from the father, then with probability *ν_a_* he is of age *a*, where $\sum_{a}\mu_{a}=\sum_{a}\nu_{a}=1$ and the units of time are entirely arbitrary. For an age-*a* mother of a newborn, let *x_a_* be the proportion of her life that, on average, she has spent as a female and, for an age-*a* father of a newborn, let *y_a_* be the proportion of his life that, on average, he has spent as a male. The long-term proportion of time spent in males and females—and thus the ratio of reproductive values (*c*_f_/*c*_m_)—will then be given by:

2.1 $$\begin{array}{c} \frac{c_{f}}{c_{m}}=\frac{\zeta \sum_{a}\mu_{a}ax_{a}+(1-\zeta )\sum_{a}\nu_{a}a(1-y_{a})}{\zeta \sum_{a}\mu_{a}a(1-x_{a})+(1-\zeta )\sum_{a}\nu_{a}ay_{a}}=\frac{\zeta T_{\mathrm{ff}}+(1-\zeta )T_{\mathrm{mf}}\;}{\zeta T_{\mathrm{fm}}+(1-\zeta )T_{\mathrm{mm}}}, \end{array}$$

where *T_ij_* is the mean time that the sex-*i* parent of a newborn has spent as sex *j*, and the units of time could be rescaled arbitrarily.

For example, in a simple dioecious species, *x_a_* and *y_a_* are simply 1, i.e. individuals spend their whole lives as the sex that they were born, and therefore *T*_fm_ = *T*_mf_ = 0. This means that in a dioecious species the ratio of average time that a gene has spent as a female versus average time spent as a male through the generations is given by

2.2 $$\begin{array}{c} \frac{c_{f}}{c_{m}}=\frac{\zeta \sum_{a}\mu_{a}a}{(1-\zeta )\sum_{a}\nu_{a}a}=\frac{\zeta }{\left(1-\zeta \right)}\frac{T_{f}}{T_{m}}, \end{array}$$

where *T*_f_ is the mean maternal age, and *T*_m_ is the mean paternal age [9,33].

Inspecting these equations, we can gauge some general patterns relating different forms of sequential hermaphroditism to the reproductive values of females and males. For instance, if a species is protogynous, then this will mean that fathers will have spent some time as females (*T*_mf_ > 0), while mothers will not have spent any time as males (*T*_fm_ = 0), which—all else being equal—serves to increase the reproductive value of females. By contrast, under protandry, mothers will have spent a fraction of their life as males (*T*_fm_ > 0), while fathers will not have spent any time as females (*T*_mf_ = 0), which—all else being equal—serves to increase the reproductive value of males.

We can further partition the reproductive values of these classes into the reproductive values of—and thus force of selection acting upon—different types of class transition. To compute these, we first need to describe the frequency of each type of class transition. For instance, the frequency of the transition from a parent of a given sex to the newborn class (i.e. the value of reproduction for that sex), will be given by the product of the frequency of the newborn class and the probability that genes in a newborn trace back to the parent of the given sex. As a gene passes through the generations, this newborn class will, on average, be encountered every $\tilde{T}$ time units, where $\tilde{T}=\zeta T_{f}+(1-\zeta )T_{m}$. This quantity, equivalent to a weighted mean parental age, is arguably the most natural measure of generation time, and when defined as such gives the result that the reproductive value of newborns is simply $c_{1}=1/\tilde{T}$ (recovering previous results found for asexuality [34]). As this class comes from females with probability *ζ*, and from males with probability 1 − *ζ*, then the relative reproductive value of reproduction through the two sexes is given by

2.3 $$\begin{array}{c} \frac{c_{f}^{r}}{c_{m}^{r}}=\frac{\zeta }{1-\zeta }. \end{array}$$

This mirrors previous results which show that it is the transmission genetics alone—and not the demography—that shape the relative ancestral contribution through reproduction made by males and females [9,33]. We may similarly calculate the reproductive value of survival by males and females, the ratio of which is given by

2.4 $$\begin{array}{c} \frac{c_{f}^{s}}{c_{m}^{s}}=\frac{\left(T_{\mathrm{ff}}-1)\zeta +T_{\mathrm{mf}}(1-\zeta \right)}{T_{\mathrm{fm}}\zeta +(T_{\mathrm{mm}}-1)(1-\zeta )}. \end{array}$$

Once again, if we assume that there is no sex change (*T*_mf_ = *T*_fm_ = 0), then we recover the results of Hitchcock & Gardner [9] as a special case ($c_{f}^{s}/c_{m}^{s}=\zeta (T_{f}-1)/((1-\zeta )(T_{m}-1)))$. Having obtained these reproductive values, we can now investigate the consequences of trade-offs between these different states and state-transitions, and, in particular, trade-offs between males and females, i.e. sexual antagonism. We first investigate the consequences for autosomal genes (*ζ* = 1/2), before considering these same trade-offs as experienced by cytoplasmic genes (*ζ* ≈ 1).

## 3. Survival and fecundity trade-offs

We now consider the conditions for a sexually antagonistic allele to be able to invade the population. For such an allele to be able to invade from rarity, its weighted marginal fitness effect when rare must be positive, where the appropriate weights upon its effects in different classes (or transitions between classes) are the reproductive values. First, we consider a sexually antagonistic allele that solely affects fecundity. This allele confers a marginal relative fecundity benefit of *σ* upon one sex, and a marginal relative fecundity cost of *τ* upon the other (table 1), with this effect equivalent across all age classes in that sex. Assuming weak selection, no population structure, and no assortative mating, then we can express the condition for a female-beneficial allele to invade as $c_{f}^{r}\sigma >c_{m}^{r}\tau$, and for a male-beneficial allele as $c_{f}^{r}\tau <c_{m}^{r}\sigma$. We may then rewrite the condition for a female-beneficial allele to invade as *τ*/*σ* < *F*, and the condition for a male-beneficial allele to invade in the form *τ*/*σ* < 1/*F*, where *F* describes the ‘potential for feminization' (cf. [11]). If *F* > 1, then the condition for invasion is less stringent for female-beneficial alleles than for male-beneficial alleles. Conversely if *F* < 1, then the condition for invasion is more stringent for female-beneficial alleles than for male-beneficial alleles. Some rearrangement reveals that for autosomal genes

3.1 $$\begin{array}{c} F=1, \end{array}$$

**Table 1.** The relationship between the fitness schemes used to plot the invasion conditions in figure 1, taken from Kidwell *et al.* [5], and the marginal relative fitness effects (*σ*, −*τ*) discussed in the main text. Marginal fitness effects are calculated in the limit of weak selection, and when the allele is vanishingly rare in the population.

	females	males
genotypic fitnesses		
*W*_00_	1	1 − *s*_m_
*W*_01_/*W*_10_	1 − *h*_f_*s*_f_	1 − *h*_m_*s*_m_
*W*_11_	1 − *s*_f_	1
marginal fitness effects		
*σ*	(1 − *h*_f_)*s*_f_	(1 − *h*_m_)*s*_m_
*τ*	*h*_f_*s*_f_	*h*_m_*s*_m_

such that under arbitrary age structure and patterns of sex change, the invasion condition is equally stringent for male-beneficial and female-beneficial alleles.

Next, we consider a sexually antagonistic allele that solely affects survival. Similar to above, this allele confers a marginal relative survival benefit of *σ* upon one sex, and a marginal relative survival cost of *τ* upon the other. Under the same assumptions as above, the condition for invasion will be $c_{f}^{s}\sigma >c_{m}^{s}\tau$ for a female-beneficial allele, and $c_{f}^{s}\tau <c_{m}^{s}\sigma$ for a male beneficial allele. We can once again rearrange the invasion conditions into a potential for feminization where $F=c_{f}^{s}/c_{m}^{s}$. Substituting in the appropriate reproductive values from above, the potential for feminization becomes

3.2 $$\begin{array}{c} F=\frac{(T_{\mathrm{ff}}-1)+T_{\mathrm{mf}}}{T_{\mathrm{fm}}+(T_{\mathrm{mm}}-1)}. \end{array}$$

As well as intersexual trade-offs between the same fitness components, we may envisage intersexual trade-offs between different fitness components. First, we consider a trade-off between female fecundity and male survival. In this case, the condition for a female-beneficial allele to invade is $c_{f}^{r}\sigma >c_{m}^{s}\tau$, and for a male-beneficial allele it is $c_{f}^{r}\tau <c_{m}^{s}\sigma$. Again, substituting in the reproductive values, the potential for feminization becomes

3.3 $$\begin{array}{c} F=\frac{1}{T_{\mathrm{fm}}+(T_{\mathrm{mm}}-1)}. \end{array}$$

We may also consider an allele that modulates a trade-off between female survival and male fecundity. In this case the conditions for a female-beneficial and male beneficial allele will be $c_{f}^{s}\sigma >c_{m}^{r}\tau$ and $c_{\text{f}}^{\text{s}}\tau \text{ < }c_{\text{m}}^{\text{r}}\sigma$, respectively. The resultant potential for feminization is

3.4 $$\begin{array}{c} F=(T_{\mathrm{ff}}-1)+T_{\mathrm{mf}}. \end{array}$$

Alongside trade-offs between sexes, we may consider trade-offs that occur within a sex. If an allele confers a fecundity benefit *σ* but a survival cost *τ*, then if it is active in females it will invade provided $c_{f}^{r}\sigma >c_{f}^{s}\tau$, and if it is active in males it will invade provided $c_{m}^{r}\sigma >c_{m}^{s}\tau$.

**Figure 1.**
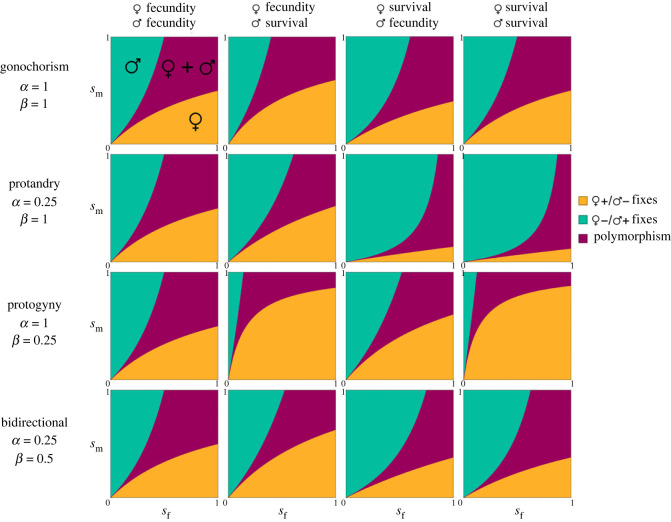
Invasion conditions for an allele affecting various types of sexually antagonistic trade-off under equal dominance. Trade-offs plotted include: fecundity in both sexes, fecundity in females and survival in males, survival in females and fecundity in males, and survival in both sexes. The various types of sexual system include: dioecy, protandry, protogyny and bidirectional sex change. The meanings of key parameters are given in table 2 and electronic supplementary material, figure S1. For all plots, we assume that *μ* = *ν* = 1/3, and *h_f_* = 1 − *h_m_*.

For the purpose of concreteness and illustration, we additionally analyse a simple population genetic model of sexual antagonism that incorporates two possible age-classes (*a* ∈ {1, 2}) and two possible sex-classes (*k* ∈ {f, m})—see electronic supplementary material, figure S1 for a visual representation of the life cycle and the associated life cycle parameters, and S3 for the model analysis. Within this structure, we allow for a variety of sex change systems by describing the following parameters: *α* is the fraction of age-2 females that came from age-1 females in the previous generation, *β* is the fraction of age-2 males that came from age-1 males in the previous generation, *μ* is the fraction of newborns that have age-1 mothers and *υ* is the fraction of newborns that have age-1 fathers. Thus, simple dioecy can be recovered by setting *α* = *β* = 1, simple protandry can be recovered by setting *α* = 0 and *β* = 1, and simple protogyny can be recovered by setting *α* =1 and *β* = 0. Using this model we may compute the various invasion conditions for different types of sexually antagonistic trade-offs in terms of the above parameters, results of which are plotted in figure 1, with the associated fitness scheme given in table 1. Additionally, expressions for the potential for feminization for various combinations of these trade-offs are given in table 2.

**Table 2.** The potential for feminization *F*, under different types of sexually antagonistic trade-offs in the two-stage model, whose life cycle is illustrated in electronic supplementary material, figure S1. *ζ* is the probability a gene copy in a juvenile was inherited from a female; *α* is the probability a gene copy in an age-2 female came from an age-1 female. *β* is the probability a gene copy in an age-2 male came from an age-1 male. *μ* is the fraction of mothers from the age-1 class; *ν* is the fraction of fathers from the age-1 class.

	male fecundity	male survival
female fecundity	$\frac{\zeta }{\left(1-\zeta \right)}$	$\frac{\zeta }{\zeta (1-\mu )(1-\alpha )+(1-\zeta )\beta (1-\nu )}$
female survival	$\frac{\zeta \alpha (1-\mu )+(1-\zeta )(1-\nu )(1-\beta )}{\zeta (1-\zeta )}$	$\frac{\zeta \alpha (1-\mu )+(1-\zeta )(1-\nu )(1-\beta )}{\zeta (1-\mu )(1-\alpha )+(1-\zeta )\beta (1-\nu )}$

## 4. Cytoplasmic genes

Thus far we have considered only autosomal genes. However, a range of important functions are encoded in genomes outwith the nucleus, for example in chloroplasts, mitochondria and other symbionts [35,36]. Moreover, such elements are interesting from the perspective of sexual antagonism as—owing to their often strictly matrilineal inheritance, and a concomitant absence of selection in relation to their male carriers—they are liable to accumulate mutations that are deleterious to males [24,25], with the converse argument applying to those cytoplasmic elements that show a primarily patrilineal mode of inheritance. The logic of this ‘mother's curse' (and ‘father's curse') has not been explored in relation to sequential hermaphrodites, wherein such cytoplasmic genes currently residing in males may still contribute to future generations even if they are never transmitted via male reproduction. Here, we investigate a broad range of cytoplasmic transmission modes, by considering an element that is inherited with probability 1 − *ζ* from a father.

Returning to our earlier invasion condition $c_{f}^{r}\sigma >c_{m}^{r}\tau$ for a (female-beneficial) sexually antagonistic allele affecting fecundity, the potential for feminization is

4.1 $$\begin{array}{c} F=\frac{\zeta }{1-\zeta }. \end{array}$$

Assuming that individuals have only one cytoplasmic type [37], then, in the limit of full matrilineal inheritance (i.e. *ζ* = 1), the potential for feminization for a cytoplasmic gene is *F* = +∞. Thus, as with conventional dioecious organisms (and other hermaphrodites), if there is strict matrilineal inheritance then cytoplasmic genes place no value upon male fecundity relative to female fecundity.

By contrast, the potential for feminization for a sexually antagonistic allele affecting survival is

4.2 $$\begin{array}{c} F=\frac{\zeta (T_{\mathrm{ff}}-1)+(1-\zeta )T_{\mathrm{mf}}\;}{\zeta T_{\mathrm{fm}}+(1-\zeta )(T_{\mathrm{mm}}-1)}, \end{array}$$

which, in the limit of strict matrilineal inheritance (*ζ* = 1), becomes *F* = (*T*_ff_ − 1)/*T*_fm_. In this case, the relative force of selection upon survival in males, relative to females, for a cytoplasmic gene ultimately becomes the average time spent in that state during a reproductive female's lifetime. This arises because even with strict matrilineal inheritance, provided there is sex change from male to female, then male survival prior to sex change may ultimately contribute to female reproduction, and thus future generations. This leads to some striking asymmetries between different types of sequential hermaphroditism. For protogynous species, where individuals are female first and male later (*T*_fm_ = 0), cytoplasmic genes will place no value upon male survival *F* = +∞. By contrast, for protandrous species, where individuals are male first and female later (*T*_fm_ > 0), cytoplasmic genes may place value upon male survival, even at a cost to the survival of females.

For trade-offs between female fecundity and male survival the potential for feminization is

4.3 $$\begin{array}{c} F=\frac{\zeta }{\zeta T_{\mathrm{fm}}+(1-\zeta )(T_{\mathrm{mm}}-1)}. \end{array}$$

Thus, as before, for a protogynous species and strict matrilineal inheritance, cytoplasmic genes place no value upon males, such that *F* = +∞, while for a protandrous species male survival is of value, such that *F* = 1/*T*_fm_. And for trade-offs between female fecundity and male survival the potential for feminization is

4.4 $$\begin{array}{c} F=\frac{\zeta (T_{\mathrm{ff}}-1)+(1-\zeta )T_{\mathrm{mf}}}{\left(1-\zeta \right)}. \end{array}$$

Thus, once again for a matrilineally inherited cytoplasmic gene (*ζ* = 1) the potential for feminization is *F* = +∞, as no genes are passed on via male reproduction. We also consider some of the within-sex life-history trade-offs in electronic supplementary material, S3.5.

## 5. Discussion

Here, we have outlined how the reproductive values of females and males are altered by different systems of sequential hermaphroditism, and thus how different sexually antagonistic trade-offs may manifest differently in these organisms as compared with dioecious species. We have shown that, regardless of the system of sex change or other sex-specific demographic parameters, the force of selection upon fecundity effects of males and females is equal—for autosomal genes—owing to their equivalent contributions to the newborn class. However, trade-offs involving survival may not be, either because the mean parental age of one sex is higher than the other, or because, with the possibility of sex change, one sex may contribute (more) to the other through survival. This results in distinct patterns of sexually antagonistic trade-offs involving survival with different forms of sequential hermaphrodites (e.g. protandry versus protogyny), and in ways that are distinct from dioecious species. Moreover, this flow of genes that occurs between the sexes as a consequence of sex change means that cytoplasmic genes, which are normally strictly matrilineally inherited, may nevertheless experience selection in males, and this yields clear-cut predictions concerning comparisons between dioecious species and different forms of sequential hermaphroditism.

While recent years have seen a renaissance in the development of theory on sexual antagonism, there has—until now—been a lack of any formal theory regarding sexual antagonism in relation to sequential hermaphrodites [18,21], in part owing to the challenges of allowing for movement of gene lineages between sexes outwith reproduction. And, although it has been suggested that trade-offs might be biased towards the first sex owing to a skewed sex ratio [22], this issue has remained unresolved. Here, we have shown more generally how patterns of sex change—varying from protandry to protogyny to bidirectional sex change—may generate biases in the force of selection towards one sex or the other, and how this varies across different fitness components (e.g. survival versus fecundity). As these patterns depend upon the direction of sex change and other demographic parameters, sequential hermaphrodites may provide excellent comparative testbeds for the theory of sexual antagonism more generally, just as they have for the theory of sex allocation [38,39]. Indeed, many systems that have previously been investigated in relation to sex allocation may also prove good systems to understand sexual antagonism. For instance, the pandalid shrimp (*Pandalus jordani*) has long been studied with regards to sex allocation [40–42]. This protandric species typically has just two breeding seasons, and shows variation among populations in the number of males that change sex, in part responding to differential harvesting pressure which alters the age distribution of the local population. These subpopulations may well show variation in both mean maternal and paternal ages, as well as variation in the timing and extent of sex change, and thus provide good systems for testing our theoretical predictions.

Not only is there little current theory regarding sexual antagonism in sequential hermaphrodites, but currently there is also a relative lack of empirical work [18,21]. This may stem from there being very few ‘classical' molecular model organisms that are sequential hermaphrodites, although new genomic tools have made molecular investigation of such non-model organisms more tractable. Combined with new theoretical developments [43–48], detection of sexually antagonistic alleles—and the signatures of sexually antagonistic selection—may be increasingly feasible in these groups. For instance, the genome of the protandrous gilthead sea bream (*Sparus aurata*) has recently been sequenced, and its sex-biased gene expressions described [49], and this may make this species, as well as other sparids, good candidates for future investigation. Additionally, one example that has previously been suggested as representing sexual antagonism is aggression in the protogynous sharpnose sandperch (*Parapercis cylindrica*) [21,50]. Here, individuals that are aggressive as females also tend to be aggressive as males later in life. However, the sex-specific optima in relation to aggression was assumed rather than demonstrated, so further investigation of other traits—such as coloration, physiology and behaviour—which are known to have sex-specific optima may be worthwhile to see whether these too reveal such correlations.

Alongside sexual antagonism, the theory we have outlined here also provides a framework with which to understand the forces shaping other life-history trade-offs, in particular senescence. The interplay between sex and senescence has recently received increasing theoretical and empirical attention [51–54] and, once again, sequential hermaphrodites provide a particularly interesting set of species and life cycles with which to investigate these phenomena, as questions about the relative value of different age classes are intrinsically bound up with questions about the value of different sexes. One particular suggestion has been that protogynous species with extreme breeding sex ratios may experience reduced senescence as, in such species, a relatively old individual might still have a great deal of reproductive potential [55]. More generally, it is interesting to consider whether, if half of the future reproduction is forced to come from above a certain age, this will shift the force of selection to relatively later in life. Conversely, it is unclear whether the reverse argument may also apply, and that by constraining much (or all) of one sex's reproduction to younger ages, then the force of selection upon older age classes is reduced relative to a comparable dioecious species. Future modelling is needed to address these questions and ensure that empirical work is best targeted to the most salient comparisons.

We have also shown how sequential hermaphroditism will alter patterns of selection in relation to maternally inherited cytoplasmic genes. While it has long been understood from a theoretical perspective that cytoplasmic and autosomal genes may differ in their interests when there are trade-offs between the sexes, e.g. ‘mother's curse' [24,25], more recently there has been an increased amount of empirical support for this effect [56,57]. We have shown here that—unlike in dioecious species—cytoplasmic genes may undergo direct selection in males, with the extent of this depending on the form of hermaphroditism considered, leading to potentially neat empirical tests. Moreover, cytoplasmic genes are expected to make different trade-offs regarding the pace of life as compared with autosomal genes. For instance, in protogynous species, mitochondria do not reproduce through the second sex, and should thus favour a faster pace of life than autosomal genes. By contrast, in protandrous species, mitochondria are expected to place a relatively greater value on reproduction later in life, and thus favour slower life histories than autosomal genes. Such intragenomic conflicts of interest are expected to be strong, and these are phenotypes for which mitochondria are likely to play important modulating roles [58].

Moreover, such cytoplasmic genes would be expected to favour not only a different pace of life from autosomal genes, but also a distinct allocation to male and female strategies. This has previously been well studied in the form of cytoplasmic male sterility in hermaphrodites (especially plants) [59–61] and male-killing endosymbionts in dioecious species (especially arthropods) [62–64]. Yet, despite the centrality of sequential hermaphrodites to sex-allocation research, little empirical or theoretical work has investigated the potential for such sex allocation distorting elements in sex-changing species. These species may reveal distinct mechanisms of sex-ratio distortion. In protogynous species cytoplasmic genes may be favoured to delay or prevent sex change, whilst in protoandrous species they may be favoured to advance sex change or alter the sex at birth.

In addition to the above reasons, it has been pointed out previously that hermaphrodites may be good study systems for sexual antagonism because they are expected to suffer more acute patterns of sexual antagonism than a comparable dioecious species [18,21] (although strong sexual antagonism may also drive the breakdown of hermaphroditism to dioecy [65,66], and thus bias the sample of extant sequential hermaphrodites). Alongside the informational constraints that dioecious species experience in uncoupling developmental pathways and physiological processes, sequential hermaphrodites—by performing both sexual strategies within the same body—are likely to have much stronger correlations between male and female traits. The specific degree of trait correlation is likely to depend greatly on the character of interest, and the particularities of the organism in question, and thus it is challenging to make generalizations. Nonetheless, we might expect those organs, tissues and traits that have high fixed costs and lower rates of turnover to be more challenging to uncouple than those that that have low fixed costs, and high rates of turnover. For example, if neuronal cells tend to last much longer than gut epithelial cells, then perhaps modifications to the gut epithelium are less constrained than remodelling the nervous system. Additionally, those tissues that are essential to maintain may also be more constrained than those that do not need to continually function; the gonads may be easier to remodel than the cardiovascular system. Similar constraints may also occur for dioecious species that are constrained to have a prolonged period before sex is determined, for example species with environmental sex determination. A better understanding of these developmental constraints may provide a further suite of productive comparative predictions in these groups.

Here, we considered conditions for invasion when fitness effects are vanishingly small. Typically, weak-selection assumptions do not matter greatly for sexual antagonism on autosomal genes (at least with non-overlapping generations), the reason being that—even under strong selection—the distribution of a sexually antagonistic allele across classes is not affected by selection in the previous generation, as alleles are reshuffled between the sexes every generation (hence issues detecting such alleles [67]). However, this will not happen for sequential hermaphrodites because—just as in age-structured populations in asymmetric genetic systems—strong selection is liable to perturb the distribution of the mutant allele across classes [18,21]. This second-order effect may potentially alter the invasion conditions for sexually antagonistic alleles, making them either more—or less—stringent depending on whether strong selection generates negative or positive associations with the class to which it confers benefits, altering the potential for polymorphism in these groups. Moreover, these patterns may well differ among different types of sequential hermaphrodites. For instance, a sexually antagonistic allele that confers fecundity benefits to females in a protogynous species will become relatively more abundant in the newborn (female) class of individuals, amplifying its benefits, while conversely a male-beneficial allele will also become more abundant in newborn females, thus dampening its benefits. Future modelling should address this, both to understand these effects in and of themselves, and to identify the systems and scenarios wherein weak selection approximations may be less accurate.

To facilitate comparison, our focus here has been on alleles that have constant proportional effects across age classes. Of course, real-world sexually antagonistic alleles are liable to have more complex phenotypic effects, where the relative magnitude of fitness effects differs across different age classes. In such cases reproductive values still provide the correct weightings to aggregate these various fitness effects; however, now one needs to treat the various age classes separately, as the magnitude of fitness effects may differ across these age classes (electronic supplementary material, S2.4). Such asymmetric fitness effects too may bias the invasion of sexually antagonistic alleles in one direction or another. Analogous to this is the analysis of the force of selection in the study of senescence, where researchers, by incorporating different mutational effects [68], or indirect fitness effects [69,70], find that the force of selection does not appear to decline with age as classically suggested [26,71,72], but may show a much more complex pattern depending on the further assumptions made. Yet, while these additional effects may shift the balance of sexually antagonistic (or antagonistically pleiotropic) alleles in particular cases, reproductive value continues to provide the underlying weightings to these effects.

We have also focused on a relatively abstract model of sex change, in which individuals are indexed solely by age and sex. This, however, ignores many of the complexities that are associated with the ecologies that shape sex change and social interactions in these groups. For instance, in many species sex change is a highly social affair, with its timing governed by the local sex ratio, and an individual's relative age, size and condition [23,38,39,73]. In the polychaete worm *Ophryotrocha puerilis* for instance, individuals are socially monogamous, and throughout the breeding season a pair of individuals may change sex repeatedly, with the larger individual being the female and the smaller the male, changing sex as their relative size changes [74]. This also may provide a good species for comparisons, as closely related species are both hermaphroditic and dioecious [75]. Moreover, alongside social interactions determining sex change, such sexually antagonistic fitness effects may also manifest through social interactions, and thus have more complex dynamics if they involve either siblings (e.g. sib-cannibalism in *Crepidula coquimbensis* [76]), or mates (e.g. sexual conflict [21]). Additionally, particular systems may involve more complicated class structure. For example, in the protandrous slipper limpet (*Crepidula fornicata*), individuals can store sperm, such that females may produce offspring with previous mating partners that exist as females at the time of the offspring's conception [77]. In these cases, sperm themselves must be separately tracked as a separate class in order not to incorrectly assign those fitness effects to males that may either have died or else become females. Building in such details will help tailor future models to particular taxonomic groups, as well as developing a better understanding of which ecological factors may drive differences in the time that genes spend in male and female bodies, the magnitude of fitness effects they may impose, and different points in the life cycle, and thus which factors and systems may be best to focus on for experimental or comparative studies of sexual antagonism.

Finally, the models we have outlined here have focused on cases where, although an individual may reproduce as either sex throughout its life, at any given point in time it is exclusively reproducing as either a female or a male. However, in reality the boundary between sequential and simultaneous hermaphroditism is porous [23]. For instance, in both *Lysmata* shrimp [78] and certain polychaete worms [79] individuals are protandric simultaneous hermaphrodites, reproducing as males when young, and then hermaphrodites when older. Additionally, many simultaneous hermaphrodites alter their sex allocation strategy as a function of their age, size and condition and environmental variables, and thus these too can be viewed as existing somewhere on the spectrum between strict sequential and strict simultaneous hermaphroditism [23]. These examples further blur the boundary between questions of sex allocation, sexual antagonism, and life-history trade-offs, and thus require richer theory to help us identify and understand the ecological factors moulding the shape and pattern of life across these diverse sexual systems.

## Data Availability

Supplementary material is available online [80].
